# Association among inflammaging, body composition, physical activity, and physical function tests in physically active women

**DOI:** 10.3389/fmed.2023.1206989

**Published:** 2023-07-18

**Authors:** Carlos Andre Freitas Santos, Gislene Rocha Amirato, Vitoria Paixão, Ewin Barbosa Almeida, Jônatas Bussador Do Amaral, Fernanda Rodrigues Monteiro, Tamaris Roseira, Yara Juliano, Neil Ferreira Novo, Marcelo Rossi, Anuska Marcelino Alvares-Saraiva, Rodolfo de Paula Vieira, Andre Luis Lacerda Bachi, Alessandro Ferrari Jacinto

**Affiliations:** ^1^Discipline of Geriatrics and Gerontology, Department of Medicine, Paulista School of Medicine, Federal University of Sao Paulo (UNIFESP), São Paulo, Brazil; ^2^Postgraduate Program in Translational Medicine, Department of Medicine, Paulista School of Medicine, Federal University of São Paulo (UNIFESP), São Paulo, Rio Grande do Sul, Brazil; ^3^Mane Garrincha Sport Education Center, Sports Department of the Municipality of São Paulo (SEME), São Paulo, Brazil; ^4^4ENT Research Lab, Department of Otorhinolaryngology-Head and Neck Surgery, Federal University of São Paulo (UNIFESP), São Paulo, Brazil; ^5^Post-graduation Program in Health Sciences, Santo Amaro University (UNISA), São Paulo, Rio Grande do Sul, Brazil; ^6^Postgraduate Program in Environmental and Experimental Pathology, Universidade Paulista, São Paulo, Brazil; ^7^Post-graduate Program in Human Movement and Rehabilitation and in Pharmaceutical Sciences, Universidade Evangélica de Goiás—Unievangelica, Anapolis, Brazil

**Keywords:** aging, inflammaging, physical activity, body composition, functional tests, cytokine profile

## Abstract

**Background:**

Inflammaging is a phenomenon that has been associated with the development and progression of sarcopenia and frailty syndrome. According to the literature, on the one side, the increase in body fat is associated with a systemic pro-inflammatory status, which consequently favors inflammaging, and on the other side, the regular practice of physical exercise can mitigate the development of this scenario. Therefore, here, we aimed to evaluate the association between inflammaging and physical factors, both body and functional, in a group of physically active older women.

**Methods:**

Seventy older women (mean age 72.66 ± 6.17 years) participated in this observational cross-sectional and were separated into the eutrophic, overweight, and obese groups. It was assessed: by bioimpedance—body fat percentage (Fat%) and total (Fat kg), skeletal muscle mass (muscle), and free fat mass both in percentage (FFM%) and total (FFMkg); by the International Physical Activity Questionnaire (IPAQ)—the time of moderate-intensity physical activity per week; by physical tests—handgrip (HG), sit-up-stand-on-the-chair in 5 repetitions (Sit-up) and vertical squat jump test (SJ); in addition to the determination of serum cytokine concentration (IL-6, TNF-α, IL-10, and IL-8), and also body mass index (BMI) and calf circumference (Calf).

**Results:**

Higher FFM% and lower body fat (both kg and %) were found in the eutrophic group than in the other groups. The eutrophic group also performed more weekly physical activity, jumped higher, and presented not only higher serum IL-6 concentration but also an increased ratio of IL-10/IL-6, IL-10/TNF-α, IL-10/IL-8 as compared to the values found in the overweight group. The obese group presented higher body fat (kg and %) and lower FFM% than the other groups and also higher serum IL-6 concentration than the overweight group. Interestingly, several significant negative and positive correlations between body composition, physical tests, and serum cytokine concentrations were found in the eutrophic and obese groups.

**Conclusion:**

While the eutrophic older women group showed a remarkable regulation of the systemic inflammatory status with positive associations in the physical parameters assessed, the overweight and obese groups presented impairment regulations of the inflammaging, which could be related to less weekly physical activity and higher body fat.

## Introduction

According to the United Nations (UN) and the World Health Organization (WHO), the decade of 2021–2030 will be committed to improving older adults' quality of life since it had been estimated that, between 2015 and 2050, the proportion of the population aged over 60 would almost double from 12 to 22% (1). The development of Collaborative Healthy Aging was based on it, but the COVID-19 pandemic imposed a new worldwide challenge, particularly to the older adult population, which was one of the populations most suffering from SARS-CoV-2 infection (2–4).

It is known that aging is a natural and heterogeneous process that, in a general way, is associated with a gradual decrease in several body system actions (5, 6). In this respect, it has been described that aged people can significantly lose their functional abilities, whether muscular, mobility, cognitive, psychological, or sensory (7). Of note, it has been highlighted that disablement in older adults can be closely related to the presence of a phenomenon called “inflammaging”, which is characterized by chronic, sterile, systemic, and subclinical low-grade inflammation, in which there is an increase in several inflammatory cytokines, such as interleukin (IL)-1, IL-6, and tumor necrosis factor-alpha (TNF-α), in addition to significant alterations in innate and acquired immunity (8, 9). In agreement with the literature, the development of inflammaging is associated with a physiological response to antigenic stress throughout life and can represent an efficient defense mechanism, especially against pathogens already known, as long as it is under control (10). Nonetheless, at the same time, the inflammaging can also be associated with a remarkable impairment in the induction of an effective defense against new antigenic challenges, which leads to this population presenting a reduction of their immune response, a well-known phenomenon observed in older adults named immunosenescence (11, 12). These phenomena, when combined, can favor the development of severe clinical situations, diseases, and syndromes common in the aged population, most notably dementia syndromes, diabetes, cardiovascular disease, cancer, osteoporosis, sarcopenia, and frailty syndrome (13–17).

Among some factors that can contribute to the inflammaging is body fat since alterations in body fat distribution and composition, specifically related to the increase in adipose tissue, a common aspect observed in older adults, promote elevation in the systemic inflammation status as a result of increased production of pro-inflammatory cytokines by infiltrated macrophages in the adipose tissues (18, 19). Raised systemic inflammation is closely associated with insulin resistance in skeletal muscle and liver, which compromises metabolic functions and increases cardiovascular risk by increasing not only circulating fatty acid levels but also lipid oxidation, which, consequently, drives an atherogenic profile (20). Beyond these aspects, it was reported that obese aged people are more vulnerable to developing sarcopenia (21) and also that a systemic pro-inflammatory status can enhance both the development and progression of this clinical condition (22, 23). In relation to sarcopenia, the European Working Group on Sarcopenia in Older People (EWGSOP), in 2019, established that sarcopenia is a muscle disease (muscle failure) characterized by reduced muscle strength, measured by using a hand dynamometer, in which values lower than 16 and 27 kg of force are considered as the cutoff for women and men, respectively, or through the sit-up-stand-of-chair test in five repetitions, in which values above 15 s are considered the cutoff. Furthermore, the EWGSOP also considered low appendicular skeletal muscle mass quantity in the diagnosis of sarcopenia, and the cutoff points with values below 15 and 20 kg, for women and men, respectively, are associated with sarcopenia occurrence (15).

To minimize the inflammaging development and progression, it has been demonstrated that the regular practice of physical exercises is a powerful tool due to its capacity to reduce body fat (24) and regulate the systemic inflammatory status, not only by the increase of the fat “burning” but also by the increase of anti-inflammatory cytokines levels, such as receptor antagonist IL-1 (IL-1ra), IL-10, and the soluble receptor of TNF-α (sTNF-R), produced during and after physical exercise performance (25–28). Another important cytokine is IL-8: This myokine is mainly produced by macrophages and endothelial cells and exerts, indistinctly, a marked chemotactic activity on leukocytes, being also an angiogenic factor (28). Therefore, analyzing the serum concentrations of these cytokines or the ratio between anti-inflammatory and pro-inflammatory ones helps to understand the adaptations of aged people in different scenarios of the aging process, especially even before becoming sarcopenic or frail (26, 29, 30). Interstingly, the literature pointed out that older adults who regularly practice physical exercises present higher numbers of naive T lymphocytes, lower circulating TNF-α levels, improvement of immune response to the influenza virus vaccination, higher muscle strength and power, and better results in physical functional tests than older adult who are non-practitioners or sedentary (30–32).

In this context, the assessment of strength, power, and balance performance provides information on the quality of the skeletal muscle and the physical conditioning of old people (6, 33, 34). In the aging process, changes occur in the quantity and quality of skeletal muscles, and physical tests can help identify vulnerabilities for the development of frailty and sarcopenia, in addition to being used in monitoring interventions aimed at improving the physical domain and the intrinsic capacity of the aged adults (35–37).

Although it is well-known that both body composition and regular practice of physical exercises can impact inflammaging development and progression (25, 26), there is still no consensus in the literature about the best model to illustrate the inflammaging repercussion within physiological senescence. Thus, in this study, we aimed to identify which factors related to body composition and physical performance could be associated with the inflammaging in physically active older eutrophic, overweight, and obese older women.

## Materials and methods

### Participants and design of the study

**Figure 1:**
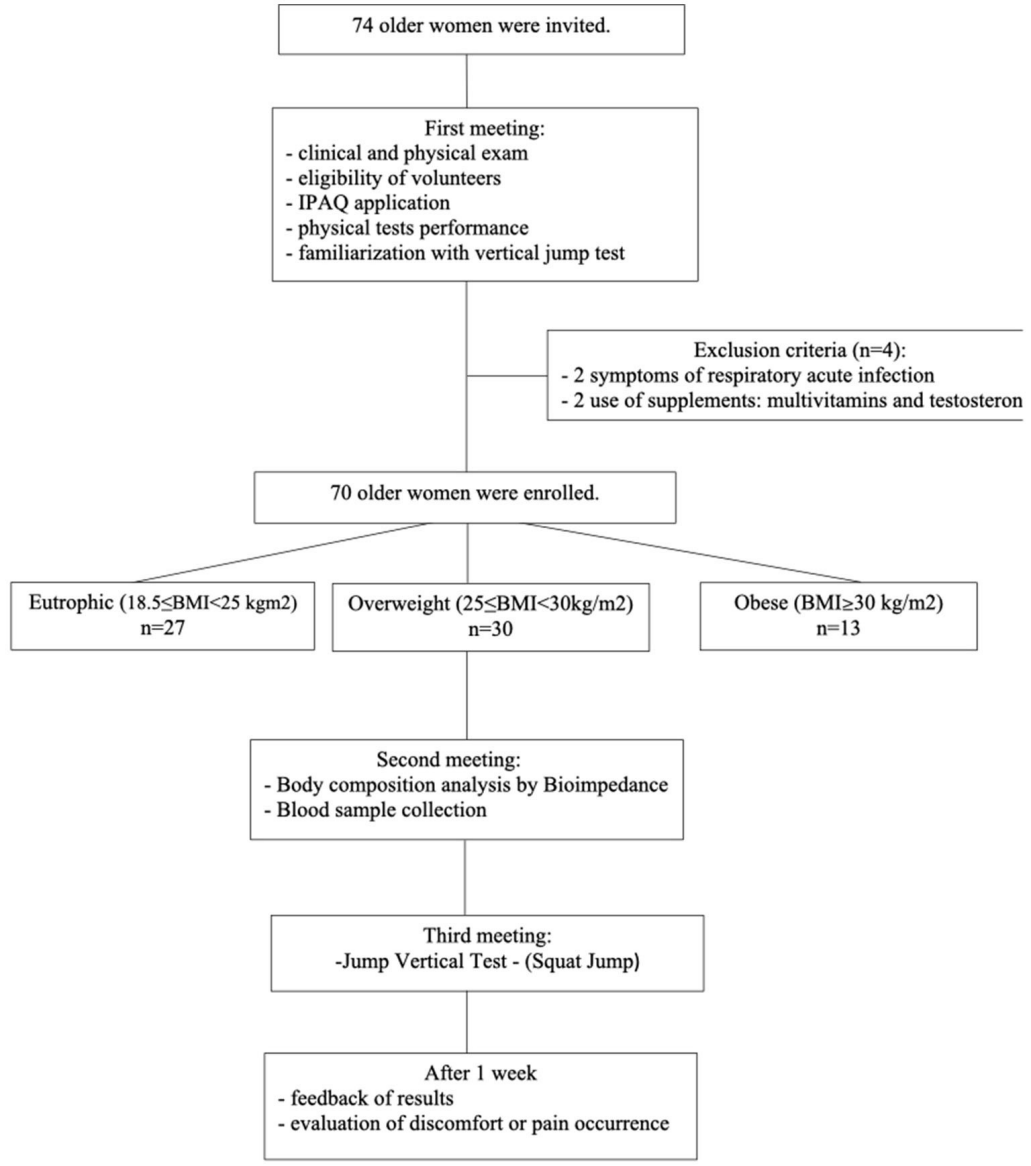
Flow diagram and study design.

In this observational cross-sectional study, initially, 74 women aged ≥ 60 years were invited to participate voluntarily between March and April 2019. However, as shown in Figure 1, 70 volunteers were included in this study since they met the inclusion and exclusion criteria as described. All volunteers were recruited from the Primary Health Care Program belonging to the Discipline of Geriatrics and Gerontology at the Federal University of São Paulo (UNIFESP). It is worth mentioning that the same geriatric physician was responsible for the clinical and physical examinations. The participants were informed of the risks and benefits of the study before data collection and gave written informed consent for their participation. They signed the informed consent form previously approved by the Ethics Committee of the Federal University of São Paulo (approval number 3.623.247) and by the National Research Ethics Committee (number CAEE:218170619.3.0 000.5505). The study was performed not only in agreement with the Ethical Standards of Exercise Practice (38) but also complied with the Declaration of Helsinki guidelines for research with humans (39).

The inclusion criteria were as follows: (i) the volunteer should regularly practice physical exercises under supervision or orientation, in the last 05 years; (ii) feel fit and have no contraindications for performing physical tests. The exclusion criteria were as follows: (i) weight change >4% in the last 12 months; (ii) use of anti-inflammatory drugs, multivitamins, protein supplements, and hormonal anabolic in the last 2 months; (iii) to present a high risk for fragility fracture in accordance to the Frax index^®^ (40); (iv) being seropositive for HIV, having neoplasms, acute or chronic infections, neurological, cardiovascular, type I diabetes mellitus, and musculoskeletal diseases that prevent physical tests.

Regarding the number of older women volunteers enrolled in the present study, it is important to cite that it was established according to sample size calculation using the G^*^Power software program (41), considering Student's *t*-test with an effect size (0.30) at α-level (0.05), the statistical power of 0.95, and also a margin of 10% losses or refusal (35). Based on it, a minimum of 70 individuals would be necessary to perform this study.

### Study design

According to the flow diagram (Figure 1), the study design was carried out in three stages. First, the volunteers previously invited to participate in this study were robust old women, without clinical alterations in the domains of intrinsic capacity (7), compensated chronic diseases, presented low nutritional risk (assessed by the Mini Nutritional Assessment—MNA) (42), adequate vision and hearing, no change in mood, preserved cognition, preserved strength, and mobility. All volunteers were submitted to clinical and physical examinations by the same geriatric physician, and four women were excluded from the study, by clinical issues.

In addition, it was also applied the International Physical Activity Questionnaire (IPAQ), the physical tests, and the familiarization with the jump test. On the same occasion, the volunteers enrolled were separated, based on BMI criteria presented by WHO (43), into three groups: eutrophic (*n* = 27, 18.5 **≤** BMI < 25 kg/m^2^), overweight (*n* = 30, 25 ≤ BMI < 30 kg/m^2^), and obese (*n* = 13, BMI ≥30 kg/m^2^). At the second meeting, it was assessed the body composition by bioimpedance and also blood sampling was carried out. After that, the third meeting was dedicated to the performance of the vertical jump test. Finally, 1 week after this last phase, all volunteers were contacted by telephone call not only to communicate the results obtained in the physical test evaluations but also to verify the occurrence of any discomfort or pain as a consequence of the performance of the vertical jump test.

### Physical activity

The level of moderate-intensity physical activity level was assessed through the International Physical Activity Questionnaire (IPAQ), validated for the Brazilian population (44, 45). By IPAQ, it is possible to estimate weekly time spent (in minutes) on moderate-intensity physical activities in different contexts of daily life (which may be characterized, subjectively, by the moderate increase in effort): (i) by physical activities of everyday life (e.g., housework); (ii) unsupervised recreational activities (e.g., dancing, cycling, running, and playing sports); and (iii) guided and supervised physical exercises (aerobics, resisted, multi-component, pilates, swimming, and water aerobics).

### Anthropometric characteristics and bioimpedance assessment

Data from body weight were carried out using a digital scale (Personal^®^ scale, Filizzola, São Paulo, Brazil) used accurately to the nearest 0.1 kg. Body height was measured using a wall-mounted stadiometer, accurate to the nearest 0.1 cm. To perform these evaluations, the women wore light clothes and no footwear. Body mass index (BMI) was calculated by the equation: weight over height squared (kg/m^2^). The left calf circumference was determined by using a measuring tape to the nearest 0.1 cm with the volunteer seated, knees bent at 90 degrees, and feet supported. The body composition was determined using the BIOSCAN 920-2-S^®^ bioimpedance equipment (Matron International Limited, UK), with the volunteer lying supine and resting (46). The bioimpedance assessment was performed in the morning with the volunteers fasting (8 h), with water allowed up to 2 h before the assessment. They were asked to empty their bladder 30 min beforehand. This evaluation was carried out in the morning with the volunteers fasting (8 h) and being allowed to drink water up to 2 h before the evaluation. Then, they were asked to empty their bladders 30 min beforehand. They were also asked not to perform physical exercises the day before, nor to drink coffee or caffeinated beverages (47). The results obtained in the bioimpedance evaluation were as follows: skeletal muscle mass (in kilograms—muscle mass kg), total body fat (in kilograms—Fat kg, and percentage—Fat %), and also fat-free mass (in kilograms—FFM kg, and percentage—FFM %).

### Physical tests

The physical tests applied in the present study were the same used in traditional protocols described in the scientific literature for older adults (35, 48). It was assessed: the sit-to-up test in the chair for five repetitions (sit-up), in which the results were expressed in seconds (s); and the handgrip (HG), in which the results were expressed in kilograms of force (kgf). Regarding the HG test, it was considered the value obtained in best performance out of three attempts, with a 1-min interval, using the dominant hand and a digital dynamometer (Jamar Hydraulic Hand Digital Dynamometer^®^, Sammons Preston Rolyan, Bolingbrook, IL, USA) (49, 50).

Concerning the evaluation of the vertical jump test, this physical test was performed on a jumping platform (Elite Jump^®^, S2 Sports, São Paulo, Brazil) (51) after the familiarization carried out during the first meeting. The digital results were expressed in centimeters (cm), which represent the height of the jump, or in watts by body mass (W/kg), which represents the vertical force exerted during the takeoff phase of the jump. Each volunteer was instructed to walk for 5 min before performing the jump tests, in order to activate the muscles of the lower limbs that would be required in this test. The volunteers performed the squat jump (SJ) modality, in which they were oriented to remain static in a knee flexion position, at an angle close to ninety degrees, for 2 s before the jump, without any preparatory movement. Five jumps were performed, with intervals of 15 s between them (35).

### Blood samples

Fasting blood samples were collected between 8 and 9 a.m. in a tube without anticoagulant compound to obtain sera aliquots. In brief, after blood coagulation, the tube was submitted to centrifugation (2,000 rpm, 4°C, 10 min), and a minimum of 500 mL of serum was added in Eppendorf's tubes that were stored at −80°C until the cytokines analyses. The volunteers were instructed not to perform physical activities of moderate or vigorous intensity in the 24 h prior to the collection.

### Cytokine determination

Cytokine concentrations were determined in the serum samples using multiplex assay (LegendPlex, Biolegend, San Diego, CA, USA) following the manufacturer's instructions. The biomarkers assessed were the tumor necrosis factor-alpha (TNF-α) and the interleukins (IL): IL-6, IL-8, and IL-10. The concentration of these cytokines was calculated using appropriate standard curves (following the manufacturer's instructions). The linearity of multiplex analysis of all cytokines assessed here was, respectively, within the range of 0–10,000 pg/mL, which includes the range of sample determinations. All correlation coefficients of standard curves were in the range of 0.93–0.99, whereas intra-assay coefficients of variance were 2–4% and interassay coefficients of variance were 7–10%. We also calculated the ratio between pro- and anti-inflammatory cytokines to evaluate the systemic inflammatory status (52).

### Statistical analysis

Initially, the data obtained in the volunteer groups were compared with the Gauss curve, and the normality of each one was determined using the Shapiro–Wilk test, followed by the evaluation of the homogeneity of variance performed through the Levene test. Parametric variables were presented as mean and standard deviation (X ± SD) and were statistically analyzed using the one-way ANOVA with Tukey's *post-hoc* test. Non-parametric variables were presented as a median and interquartile range, and it was statistically analyzed using the Kruskal–Wallis test with Dunn's *post-hoc* test. A multivariate regression analysis was used to determine the influence of body composition and physical performance variables on the systemic inflammatory status, assessed by serum concentration of both pro- and anti-inflammatory cytokines. Pearson's or Spearman's correlation coefficient analysis was used to assess the association between the parameters obtained in the volunteer groups. The significance level was set at 5% (*p* < 0.05).

## Results

Table 1 presents the anthropometric and physical characteristics of the volunteers enrolled in the present study. Based on the one-way ANOVA with Tukey's *post-hoc* test, the statistical analysis of these characteristics in the eutrophic, overweight, and obese groups showed that (1) the group with overweight performed less minutes of moderate-intensity physical activity than the eutrophic group; (2) whereas the BMI, calf circumference, and body fat (both in absolute and relative values) values were lower, the FFM values (both in absolute and relative values) were higher in the eutrophic group than the values found in the other volunteer groups; and (3) overweight group presented lower BMI values and a higher percentage of body fat and FFM than the obese group, as well as a higher muscle mass values than the eutrophic group.

**Table 1:** Mean and standard deviation (X_SD) of age, physical activity time, anthropometric characteristics (BMI and CALF), and body composition (body fat kg, body fat%, muscle mass, FFM kg, and FMM%); as well as the results obtained in the statistical analysis between the volunteers separated into eutrophic, overweight, and obese groups, according to their BMI values.

**Variable**	**All women**	**18.5 ≤ BMI < 25 (a)**	**25 ≤ BMI < 30 (b)**	**BMI ≥ 30 (c)**	**P-value**
	***n*** = **70**	***n*** = **27**	***n*** = **q0**	***n*** = **13**	
Age (year)	72.66 (±6.17)	72.52 (±6.13)	73.30 (±5.68)	71.46 (±7.56)	n.s
Physical activity time (min/week^*^)	578 (±583)	822 (±722)^§^	408 (±409)	439 (±412)	^§^*p* = 0.019
BMI (kg/m^2^)	26.46 (±4.18)	22.5 (±1.48)^§, #^	27.17 (±1.48)^†^	33.00 (±2.75)	^§^*p* < 0.001 # *p* < 0.001 ^†^*p* < 0.001
Calf circumference ( cm )	35.25 (±2.82)	33.72 (±2.41)^§, #^	36.11 (±2.47)	36.80 (±3.06)	^§^*p* = 0.009 ^#^*p* = 0.020
Fat (kg)	24.82 (±8.86)	17.15 (±3.50)^§, #^	26.58 (±5.10)^†^	38.44 (±5.62)	^§^*p* < 0.001 #*p* < 0.001 ^†^*p* < 0.001
Fat (%)	39.24 (±8.25)	32.66 (±5.15)^§, #^	40.8 (±5.33)^†^	50.84 (±5.02)	^§^*p* < 0.001 #*p* < 0.001 ^†^*p* < 0.001
Muscle mass (kg)	18.13 (±2.74)	17.36 (±2.67)^§^	18.86 (±2.29)	18.12 (±3.61)	^§^*p* = 0.030
FFM (kg)	37.01 (±5.84)	35.67 (±5.8)	38.56 (±5.6)	36.34 (±6.0)	n.s.
FFM (%)	61.09 (±8.0)	67.41 (±5.07)^§, #^	58.99 (±5.41)^†^	50.31 (±5.56)	^§^*p* < 0.001 ^#^*p* = 0.005 †*p* < 0.001

^*^These values were obtained by using IPAQ (International Physical Activity Questionnaire); BMI, body mass index; FFM, fat-free mass. Significances: ^§^to a-b; ^#^to a-c; ^†^to b-c; n.s, no significant.

Table 2 shows the descriptive values of the physical tests observed in the volunteer groups. Using the one-way ANOVA analysis with Tukey's *post-hoc* test, it was found that higher values in the vertical jump test performance presented as the average of the height reached in the best jump (in centimeters), in the eutrophic group than the values observed in the overweight group. Interestingly, on average, the eutrophic group presented less strength (expressed in watts per kilogram of corporal mass) than the other volunteer groups (overweight and obese) to perform the vertical jump test. In addition, the obese group showed, on average, higher strength to perform this physical test than the values observed in the overweight group. No significant difference was found in the other physical tests assessed here. It is noteworthy to mention that, in accordance with the results obtained in the physical tests and muscle mass measure, none of the older women who participated in the present study presented sarcopenia, based on the criteria proposed by EWGSOP, formerly cited (15).

**Table 2:** Mean and standard deviation (X_SD) obtained in the physical tests (HG, Sit-Up, SJ cm, SJ W/kg). In addition, the significant differences obtained in these parameters in the volunteers were separated into eutrophic, overweight, and obese groups.

**Variable**	**All women**	**18.5 ≤ BMI < 25 (a)**	**25 ≤ BMI < 30 (b)**	**BMI ≥30 (c)**	**P-value**
	***n*** = **70**	***n*** = **27**	***n*** = **30**	***n*** = **13**	
HG (kgf)	22.16 (±4.6)	21.79 (±3.28)	22.17 (±4.77)	22.88 (±6.56)	n.s
Sit-up (s)	9.86 (±2.73)	9.34 (±2.37)	10.13 (±3.05)	10.37 (±2.71)	n.s
SJ (cm)	11.61 (±4.14)	13.29 (±4.59)^§^	10.65 (±3.18)	10.33 (±4.22)	^§^*p* = 0.014
SJ (W/kg)	22.62 (±5.04)	20.52 (±5.9)^§, #^	23.15 (±3.66)^†^	25.75 (±3.8)	^§^*p* = 0.048 ^#^*p* = 0.007 ^†^*p* = 0.042

HG, handgrip; Sit-up, sit-to-up test in the chair for five repetitions; SJ, squat jump. Significances: ^§^ to a-b; ^#^ to a-c; ^†^ to b-c; n.s, no significant.

**Figure 2:**
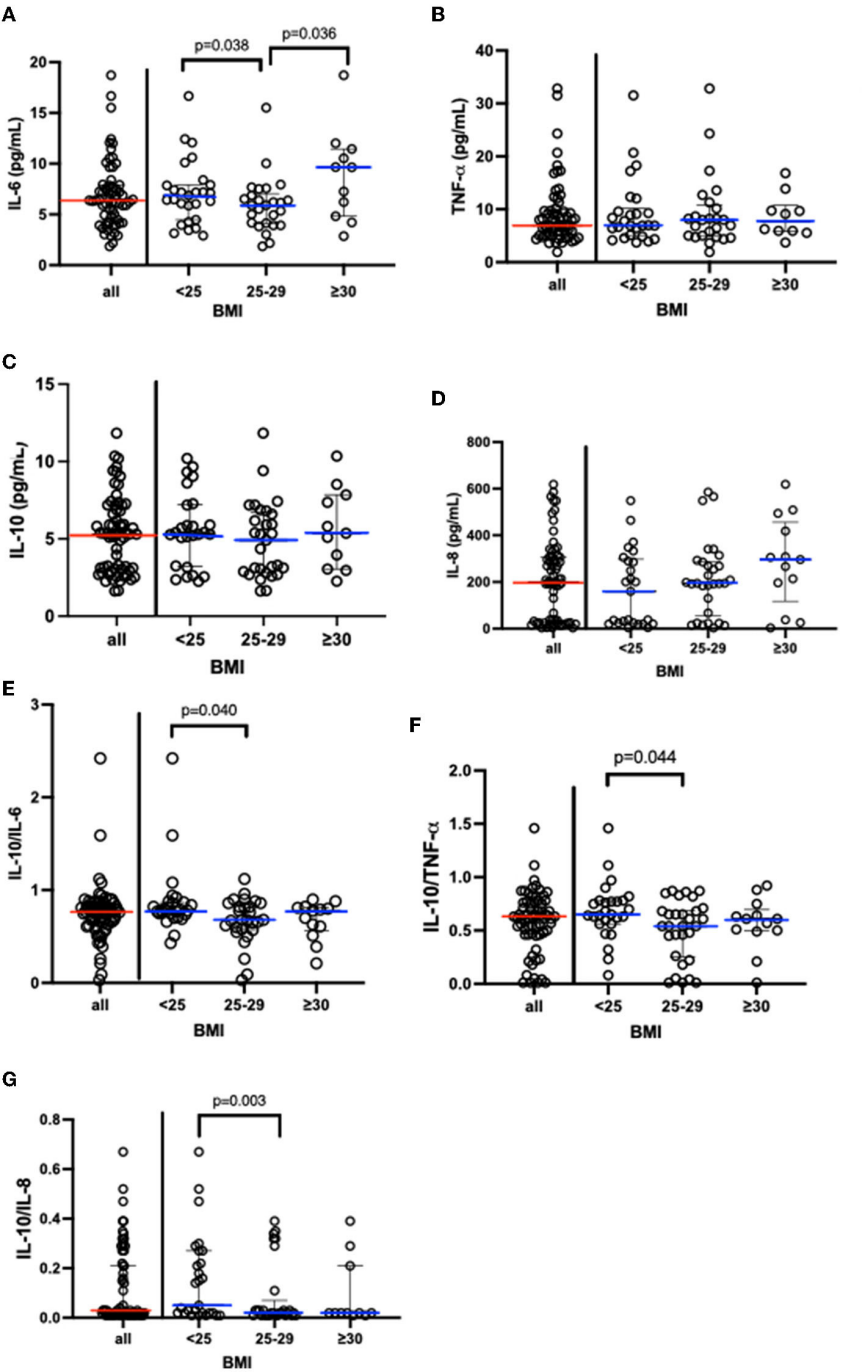
Results [median and interquartile range (X_25–75)] concerning of systemic cytokine concentration **(A)** IL-6, **(B)**TNF-α, **(C)** IL-10, **(D)** IL-8; and also the ratio between **(E)** IL-10/IL-6, **(F)** IL-10/TNF-α, **(G)** IL-10/IL-8. In addition, the data obtained when the volunteers were separated into eutrophic, overweight, and obese groups. IL, interleukin; BMI, body mass index; TNF-α, alpha tumor necrosis factor.

Figure 2 shows not only the results obtained in the systemic cytokine analysis but also the value of the ratio between the pro- (IL-6, TNF-α, and IL-8) and anti-inflammatory cytokines (IL-10), presented in the median and interquartile range, in the volunteer groups (eutrophic, overweight, and obese). Lower systemic levels of IL-6 (Figure 2A) were found in the overweight group than the values observed in the eutrophic and obese groups. No other significant difference was found in TNF- α (Figure 2B), IL-10 (Figure 2C), and IL-8 (Figure 2D) values between the volunteer groups. Based on the ratio analysis, a significant reduction of the ratios was found between IL-10/IL-6 (Figure 2E), IL-10/TNF-α (Figure 2F), and IL-10/IL-8 (Figure 2G) in the overweight group as compared to the values observed in the eutrophic group. The values obtained in the analysis of the systemic cytokine concentrations in the volunteer groups are presented in Supplementary Table S1.

Table 3 shows the results of the multiple linear regression analysis with adjustment for the time of physical activity, anthropometric and body composition measurements, and physical tests, as well as the cytokine analyses in the volunteers who participated in this study, regardless of the BMI values. It was observed negative associations between the time of moderate-intensity physical activity in the week and IL-8 values, between the percentage of body fat and the IL-10/IL-6 ratio, between the free fat mass (in absolute values) and the IL-10/IL-8 ratio, and between vertical jump (cm) test and TNF-α. Conversely, positive associations were found between the time of moderate-intensity physical activity in the week and TNF-α values, between the percentage of fat-free mass and IL-10 values, between IL-6 and handgrip or vertical jump (cm) tests, and between IL-8 and vertical jump (cm) test.

**Table 3:** Significant results obtained in the multiple linear regression analysis between the parameters related to the physical activity time, anthropometric measurements, body composition measurements and the physical tests or the cytokine profile assessed in all volunteers enrolled in the study.

**Variables**	**ß-value**	**95% CI**	**P-value**	***R* ^2^**
Physical activity time^*^–TNF-α	0.009789	0.002813 to 0.01676	0.007	0.872
Physical activity time^*^–IL-8	−0.008502	−0.01541 to −0.001596	0.017	0.797
Fat %—IL-10/IL-6	−16.92	−31.95 to −1.891	0.028	0.815
FFM kg—IL-10/IL-8	−29.47	−54.21 to −4.739	0.021	0.922
FFM%—IL-10	0.3489	0.05762 to 0.6401	0.020	0.990
HG—IL-6	0.03975	0.003141 to 0.07636	0.034	0.915
SJ cm—IL-6	0.04248	0.009323 to 0.07564	0.013	0.914
SJ cm—TNF-α	−0.01333	−0.02553 to −0.00112	0.033	0.872
SJ cm—IL-8	0.01217	0.0001551 to 0.02419	0.047	0.802

^*^These values were obtained by using IPAQ (International Physical Activity Questionnaire); FFM, fat-free mass; HG, handgrip; SJ, squat jump; IL, interleukin, TNF-α, alpha tumor necrosis factor.

**Figure 3:**
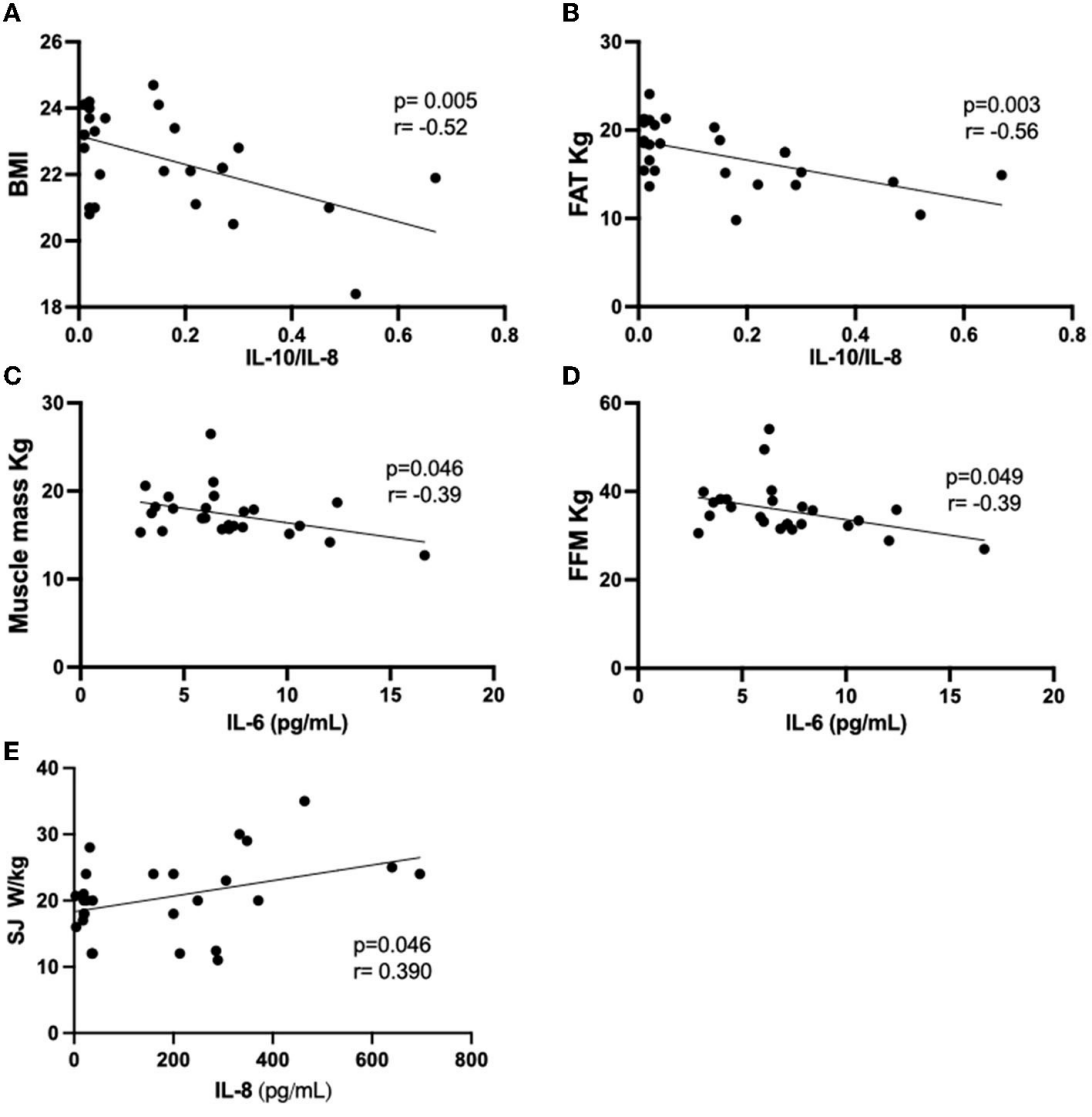
Results of the Pearson's coefficient correlation analysis of anthropometric or body composition characteristics with cytokines or cytokine ratio in the eutrophic group. **(A)** BMI AND IL-10/IL-8; **(B)** FAT kg and IL-10/IL-8; **(C)** Muscle mass and IL-6; **(D)** FFM and IL-6; **(E)** SJ W/Kg and IL-8. IL, interleukin; BMI, body mass index; FFM, fat-free mass; SJ, squat jump.

**Figure 4:**
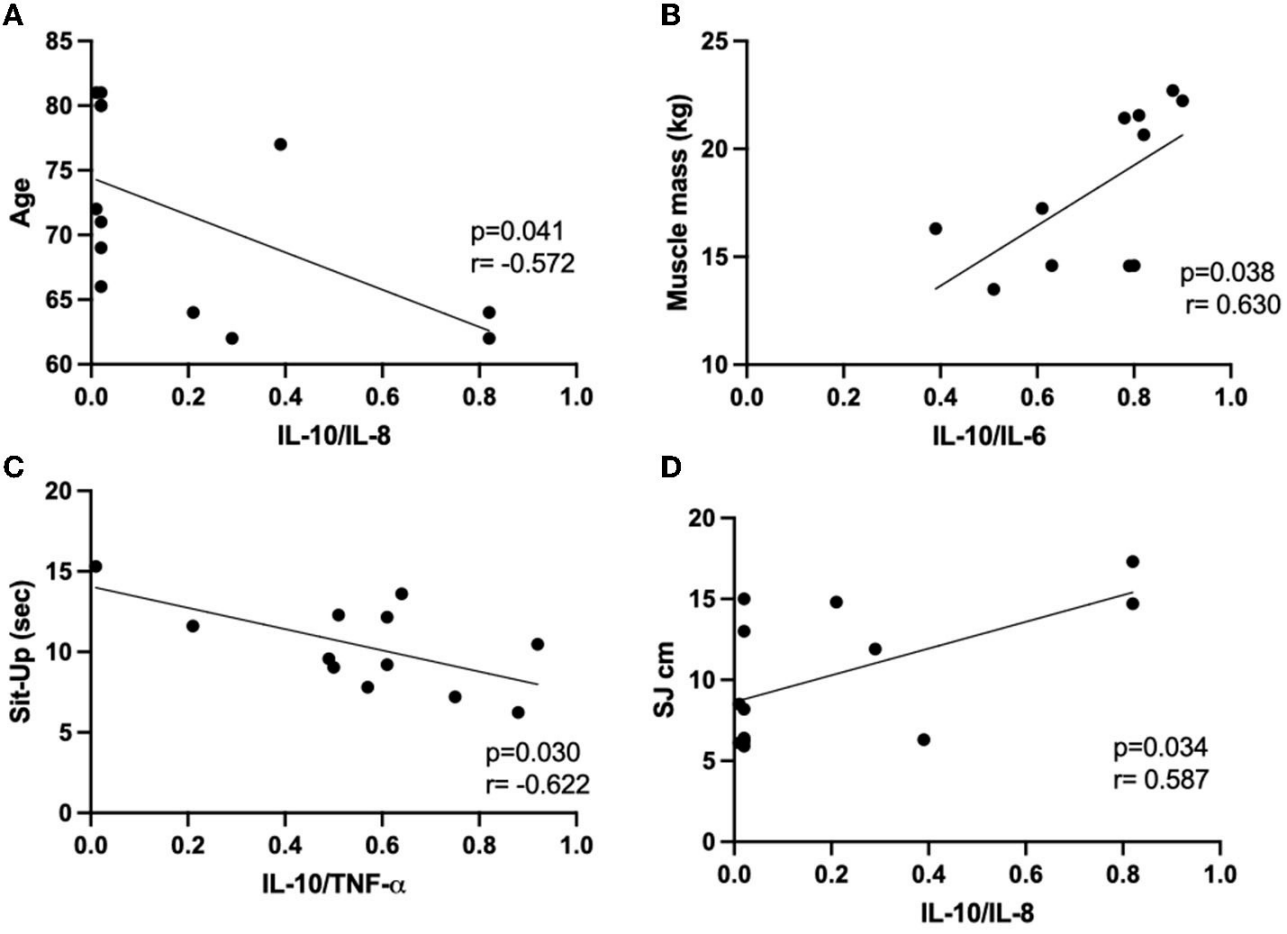
Results of the Pearson's coefficient correlation analysis of anthropometric or body composition characteristics with cytokines or cytokine ratio in the obese group. **(A)** age and IL-10/IL-8; **(B)** muscle mass and IL-10/IL-6; **(C)** sit-up and IL-10/TNF-α; **(D)** SJ cm and IL-10/IL-8. IL, interleukin; Sit-Up, sit-up test in the chair for five repetitions; TNF-α, alpha tumor necrosis factor; SJ, squat jump.

Since significant associations were found in multiple linear regression analysis between anthropometric and body composition, as well as physical tests and the cytokines assessed here, we followed the correlation analysis between these data obtained in each volunteer group. Figures 3, 4 present the significant results found in this analysis in eutrophic and obese groups, respectively. There were no significant correlations in the overweight group.

In the eutrophic group (Figure 3), it was found negative correlations between the IL-10/IL-8 ratio and BMI (Figure 3A) or body fat (in kg, Figure 3B), as well as between IL-6 values and muscle mass (in kg, Figure 3C) or FFM (in kg, Figure 3D). Conversely, a positive correlation was found between the vertical jump test (W/kg) and IL-8 (Figure 3E). Concerning the results obtained in the obese group (Figure 4), negative associations were found between the values of age and the IL-10/IL-8 ratio (Figure 4A) and also between the values of the sit-up test and the IL-10/TNF-α ratio (Figure 4C). On the other hand, positive correlations were found between the muscle mass (in kg) and IL-10/IL-6 ratio (Figure 4B) and between the vertical jump test (in cm) and the IL-10/IL-8 ratio (Figure 4D). Supplementary Table S1 presents the correlations between the minutes by the week of moderate physical activities, assessed by IPAQ, and the values of systemic cytokine concentration or their ratios in the eutrophic, overweight, and obese groups. It was possible to observe that there were no significant associations between them.

Concerning the evaluation of the volunteers' physical safety in this study, falls and physical accidents during their performances were not verified. In addition, contact was made by telephone 1 week following the performance test, and we verified that there was no occurrence of discomfort or pain related to vertical jump tests.

## Discussion

First of all, it is essential to highlight that the older women who participated in the present study performed on the time of moderate-intensity physical activity, expressed in minutes per week, above the minimum value recommended by the WHO (53), as well as that the clinical evaluations, in association with body composition and physical tests analysis, showed that none of the volunteers had sarcopenia, following the criteria proposed by the EWGSOP (15).

It is utmost of importance to point out that, until now, there are no references that allow classifying “normal” or “health” concentrations of systemic cytokines, particularly in the older adult population (25, 26). Thus, we opted to compare our results with the data available in the literature. For instance, compared with the data presented in the study performed by Lavin et al., which evaluated the serum concentrations of the cytokines in a group of eutrophic older adults who performed combined-exercise training (both aerobics and resistances exercises) and in a group with healthy non-exercisers older adults, the basal values (pg/mL) of IL-6 were 2.0 ± 0.2 and 3.9 ± 1.2, and the basal values of TNF-α were 1.7 ± 0.2 and 1.3 ± 0.2, respectively (54), which were different from the values found in the present study (Supplementary Table S1). Conversely, the serum IL-6 concentration (pg/mL) presented by a healthy population of British women over 50 years was 5.67 ± 2.02 (55), which is similar to the values observed in this study. Beyond these data found in healthy individuals, in studies that evaluated older adults with clinical conditions of evident inflammatory vulnerability, the values of systemic cytokines concentration were different from our findings. For instance, octogenarians admitted with a hip fracture at a hospital in Amsterdam presented 1.61 (1.08–2.42, pg/mL) of serum IL-8 concentration (56), whereas, in an Italian study, the serum mean values of IL-8 and IL-10 (pg/mL), in 18 seniors admitted in a hospital for elective surgeries, were 10.8 ± 4.4 and 5.3 ± 3.7, respectively (57). Moreover, Brazilian men aged 60–80 with systemic arterial hypertension and type 2 diabetes mellitus, both eutrophic and overweight, had serum IL-10 concentrations (pg/mL) with mean values of 90.13 ± 24.37 and 64.34 ± 23.81, TNF-α concentrations of 1.49 ± 0.378 and 3.01 ± 0.448, and IL-10/TNF-α ratios of 8.38 ± 3.06 and 32.49 ± 18.81, respectively (29). In another study, an adult Spanish women group with symptomatic fibromyalgia submitted to a physical exercise program showed, before the intervention, a serum IL-8 concentration of 157 ± 35 pg/mL (58), which were values similar to find in the present study. Hence, in conjunct, these pieces of information corroborate the previous mention that, until now, there are no reference values for the concentrations of the systemic cytokines, which allows us to define “normal” or healthy condition for the older adult population.

Regarding inflammaging development, this phenomenon is closely associated with body fat (12–14) and, interestingly, can be mitigated by regular practice of physical exercise (9, 10). Based on it, to better assess these aspects in an older women population, we grouped the volunteers into three subgroups, based on the WHO body mass index classification (43), and several clinical parameters related to anthropometry, body composition, and physical tests were evaluated. As expected, elevations in body fat values were found in the groups with higher BM, in contrast to the reductions in the FFM percentages when the BMI increases. Interestingly, the overweight group presented a higher muscle mass, in absolute values (kg), than the values found in the eutrophic group. Corroborating this finding, it was reported in the literature that people with raised body fat also showed an increased amount of skeletal muscle mass (59). In this respect, it had been suggested that this associative aspect is related to the fact that these people perform their simple day-to-day tasks, or daily physical activities in a similar way to strength training (60), but with infiltrated fat in their muscular architecture (61, 62).

Beyond these observations, we also observed that the calf circumference in the eutrophic group was lower than the values found in the other volunteer groups. Although calf circumference has been associated with skeletal muscle reserve (62, 63), our finding that the eutrophic group performed better in the vertical jump test, specifically the squat jump (SJ), reaching greater heights (in cm), in association with the lower values found in the power (W/kg) applied to perform this physical test, particularly in comparison with the overweight group, can putatively indicate that the increased time dedicated to the regular practice of physical exercise, observed in this group, could be useful to optimize their performance.

Another point that needs to be cited is related to the observation that the eutrophic group showed increased systemic IL-6 concentration as compared to the values found in the overweight group. Although we cannot affirm, this difference could be putatively associated with the higher time of moderate-intensity weekly physical activity reported by the eutrophic group (2-fold more) since it was reported that IL-6 can be acutely produced during exercise training and it can act as a molecule implicated in the energy metabolism regulation and indirectly mediate an anti-inflammatory effect by enhancing the IL-10 levels (64–66). Based on its prominent capacity to regulate energy metabolism, we can suggest that the higher IL-6 concentration in the eutrophic group could be useful to maintain their anthropometric and body composition since they presented less body fat values and a higher percentage of fat-free fraction as compared to the values found in the overweight group. Furthermore, this higher IL-6 concentration found in the eutrophic group allows us to also suggest that a systemic anti-inflammatory status was generated since this group showed an increased ratio between IL-10, a classical anti-inflammatory cytokine, and the IL-6, TNF-α, and IL-8, all classified as pro-inflammatory cytokines as compared to the values observed in the overweight group, as previously reported in the literature (25, 29).

Conversely, the elevated and chronic maintenance of systemic IL-6 concentration is closely implicated in several unhealthy endpoints, particularly favoring the development of diseases and comorbidities (25, 67, 68). According to the literature, one of the possible sites of IL-6 production is the adipose tissue, which is mainly associated with obesity development (10, 12, 69). These pieces of information can support our findings, in which the obese group presented increased systemic IL-6 concentration that could be derived from their higher mass fat (~40 and 25% in absolute and relative values, respectively) as compared to the values found in the overweight group. Beyond these differences, interestingly, we also observed that the average vertical jump values measured in terms of the ratio of the power (W) to body mass (kg) were higher in the obese group, demonstrating that they were able to generate greater vertical force in a fraction of a second in the takeoff of the squat jump (35, 70), even though they did not achieve the best performance, assessed by the jump height (in cm). Similarly, when comparing the overweight and eutrophic groups, the former exerted greater power and worse performance (at the height of the jump) in performing the vertical jump. Interestingly, the overweight women had a greater amount of muscle mass than the eutrophic women. These results can reinforce the importance of assessing muscle power in the older adult population, even though the fact that aged people with higher BMI presented a worse performance in the vertical jump height is not a novelty (35, 71). However, until now, it has been not reported that older women with overweight and who regularly practice physical exercises are able to generate greater vertical force and worse performance at jump height than eutrophic women. In this respect, the suggestive explanations for these data obtained in the overweight and obese group could be summarized in two points: (i) during physiological aging, these individuals showed an increase in fatty infiltration in the skeletal muscles, which accentuates the worse quality of muscle fiber contraction and the loss of muscle strength and power, resulting in a reduction in movement performance (61, 72, 73) (ii) and that this condition drives a chronic adaptation in which older adults with more body mass generate a greater amount of fast-twitch muscle fibers (type II fibers) that allowed them to exercise more strength and muscle power to perform their daily activities (60, 74).

Other remarkable findings reported here were obtained through the multiple linear regression analysis. Based on these data, it was possible to prove that the best adaptations to the inflammaging, assessed by the cytokine concentration, were associated with more weekly time dedicated to moderate-intensity physical activity, a lower percentage of body fat, a higher percentage of fat-free mass, and better performance in the physical tests, both handgrip and in the vertical jump test (maximum height in cm). Moreover, the result of a negative association between physical activity time in moderate intensity and IL-8, a pro-inflammatory cytokine, reinforces the literature, indicating that physical exercise is able to favor the generation of a systemic anti-inflammatory status (69, 75). It is also worth mentioning that, in agreement with the studies, elevations in the IL-8 during physical exercise performance can be involved not only in an inflammatory response elicited by muscle contraction (28, 76) but also can be related to angiogenic phenomena that can improve the circulation in the muscle tissue (77). Another negative association found was between the values of FFM (in kg) and the IL-10/IL-8 ratio, which could indicate that a rise in fat-free mass would be associated with an increase in IL-8 level, corroborating the idea that this interleukin is associated with the emergence of new capillary blood vessels in active skeletal muscle, however, remembering that this cytokine can depict a pro-inflammatory status too (76, 78).

Regarding the aging process, the scientific literature has suggested that the follow-up of adult athletes or even individuals who maintain a regular practice of physical exercises throughout life can be the best way to assess the physiological process of aging (26, 63, 78).

Based on it, the results obtained in the older adult population who participated in this study could be useful to demonstrate some significant aspects of aging, mainly associated with inflammaging. Particularly, the results obtained in the intragroup correlation analysis of the eutrophic group showed significant negative associations between systemic IL-6 concentration and muscle mass or FFM (absolute values in kg), as well as between the IL-10/IL-8 ratio and BMI or body fat (absolute values in kg), which together reveals that the better anthropometric and body composition was associated with a prominent regulation of the systemic inflammatory status. In addition, it was also observed another significant positive correlation between IL-8 and jump power, which can indicate that better performance in the vertical jump test is associated with this inflammatory molecule that can act as an important cytokine involved in the angio-protection of skeletal muscle, as formerly cited (28, 77).

In the obese group, it was observed that the lower weekly time dedicated to physical activity was associated with a higher percentage of body fat and a lower percentage of FFM. Furthermore, negative correlations between age and IL-10/IL-8 and also between the sit-up test (in seconds) and IL-10/TNF-α, as well as positive correlations between muscle mass (absolute values in kg) and IL-10/IL-6 and also between vertical jump and IL-10/IL-8 were found and together can indicate that a regulated systemic inflammatory status is essential to achieve a better performance in some physical tests and improve skeletal muscle mass. Even though the obese group had presented an increase in their systemic IL-6 concentration, which chronically can increase the vulnerability to unhealthy clinical outcomes related to inflammaging (13, 15), our data corroborate the expectation that among older women with obesity, the body fat loss should be desirable (8, 9, 25) and also that better control of inflammatory status can positively impact on physical activity performance.

It is paramount to point out that the absence of significant correlations in the intragroup analysis in the overweight group could be putatively attributed to the fact that this group is composed of older women with adaptive characteristics closer to eutrophic or obesity, which led us to obtain very discrepant results and no significant associations could be found.

In the same way, despite the multivariate analysis showing, in general, positive correlations between higher weekly time dedicated to performing moderate-intensity physical activity and better inflammatory adaptations, in an interesting way, the correlation analysis of volunteer groups did not show statistically significant values between the physical activity time in moderate intensity and the absolute cytokines values or cytokines ratio (Supplementary Table S2), which could be putatively associated with a possible heterogeneity of the results found in the volunteer groups.

### Limitations of the study

In the present study, we can highlight as limitations of the study: (1) the lack of results concerning the abdominal circumference, which could be used to better characterize the volunteer groups; (2) the lack of a sedentary group, which could allow us to compare our results and improve our understanding about the inflammaging; (3) even though the IPAQ, used in this study, allowed us to measure the different ways of performing daily physical activities of moderate intensity performed by the volunteers, the use of more technological tools, such as the pedometer and global positioning system (GPS), could improve the measurement of physical activities and occupational factors that could impact on weekly energy expenditure; (4) similarly, despite the clinical evaluation and use of the MNA allowed us to rule out nutritional risk in the volunteers, a more assertive assessment of dietary habits could add relevant information about the nutritional profile of the older adults participating in this study.

## Conclusion

Based on the results obtained in this study, we can suggest that (1) long-standing physical exercise performed by older women can favor the regulation of the systemic inflammatory status, which was associated with some positive effects in the anthropometric, body composition, and physical tests; (2) the eutrophic group presented the better physical parameters than overweight (more time of moderate-intensity physical activity, lower amount of body fat, and better performance in vertical jump height), which can be putatively associated with pivotal adaptations of inflammaging (expressed here in terms of pro- and anti-inflammatory cytokine associations); and (3) the obese group presented lower regulation of systemic inflammatory status compared to the overweight group, which can be associated with higher body fat. Further studies including older adult populations with different intrinsic capacities are necessary to improve our understanding of the inflammaging phenomenon.

## Data availability statement

The raw data supporting the conclusions of this article will be made available by the authors, without undue reservation.

## Ethics statement

The studies involving human participants were reviewed and approved by Ethics Committee of the Federal University of Sáo Paulo (approval number 3.623.247) and by the National Research Ethics Committee (number CAEE:218170619.3.0 000.5505). The patients/participants provided their written informed consent to participate in this study.

## Author contributions

CS is responsible for managing the study and writing of the article and he was the doctor responsible for the physical and clinical evaluations of the volunteers. GA was responsible for training and physical test familiarization. VP, EA, and JD contributed equally in the collection of blood samples and dosage of interleukins in the blood. FM and TR contributed equally in the evaluation of bioimpedance and performance of physical tests. YJ and NN contributed equally to article writing and literature review. MR and AA-S contributed equally to the statistical analyzes. RV contributed to the writing and revision of the English language. AB and AJ contributed equally to conception and design of the study. All authors contributed to the article and approved the submitted version.
