# Supplementary material for: Association among inflammaging, body composition, physical activity, and physical function tests in physically active women
Source: Front Med (Lausanne). 2023 Jul 18;10:1206989. doi: 10.3389/fmed.2023.1206989 (PMC10390738; doi:10.3389/fmed.2023.1206989)
Supplement: Supplementary file 1 [file Table_1.docx]

| **Variable** | **All women**  **n=70** | **18.5≤BMI<25 (a)**  **n=27** | **25≤BMI<30 (b)**  **n=30** | **BMI**$\boldsymbol{\geq}$**30 (c)**  **n=13** |
| --- | --- | --- | --- | --- |
| IL-6 (pg/mL) | 6.47  (4.65-9.65) | 6.85  (4.48-7.90) | 5.91  (4.09-7.04) | 9.64  (4.82-11.44) |
| TNF-a (pg/mL) | 8.22  (5.75-14.61) | 6.95  (5.06-10.20) | 7.98  (4.88-10.74) | 7.68  (5.83-10.78) |
| IL-10 (pg/mL) | 5.35  (3.10-7.20) | 5.40  (3.24-7.27) | 5.10  (2.86-6.82) | 5.8  (3.50-9.43) |
| IL-8 (pg/mL) | 199.2  (25.37-306.40) | 159.70  (20.12-305.8) | 194.00  (56.01-287.60) | 296.00  (117.00-456.50) |
| IL-10/IL-6 | 0.77  (0.64-0.86) | 0.78  (0.74-0.87) | 0.68  (0.57-0.85) | 0.78  (0.56-0.82) |
| IL-10/TNF-$\alpha$ | 0.63  (0.47-0.76) | 0.65  (0.56-0.78) | 0.54  (0.25-0.69) | 0.61  (0.49-0.69) |
| IL-10/IL-8 | 0.03  (0.02-0.27) | 0.50  (0.02-0.27) | 0.02  (0.01 to 0.16) | 0.02  (0.02 to 0.34) |

Table S1. Results [median and interquartile range (X_25-75)] concerning of systemic cytokine concentration (IL-6, TNF-a, IL-10, IL-8) and also the ratio between IL-10/IL-6, IL-10/ TNF-α, IL-10/IL-8. In addition, the data obtained when the volunteers were separated into eutrophic, overweight, and obese groups.

Note: IL, interleukin; BMI, body mass index; TNF-α, alpha tumor necrosis factor.
