# Supplementary material for: Association among inflammaging, body composition, physical activity, and physical function tests in physically active women
Source: Front Med (Lausanne). 2023 Jul 18;10:1206989. doi: 10.3389/fmed.2023.1206989 (PMC10390738; doi:10.3389/fmed.2023.1206989)
Supplement: Supplementary file 2 [file Table_2.docx]

| **Variables** | **18.5≤BMI<25** | **25≤BMI<30** | **BMI**$\boldsymbol{\geq}$**30** |
| --- | --- | --- | --- |
| IL-6  x  IPAQ min/week | p= 0.448  r= -0.153 | p= 0.480  r= -0.139 | p= 0.567  r= 0.184 |
| TNF-α  x  IPAQ min/week | p= 0.463  r= -0.147 | p= 0.963  r= -0.009 | p= 0.052  r= 0.572 |
| IL-10  x  IPAQ min/week | p= 0.350  r= 0.187 | p= 0.977  r= 0.006 | p= 0.433  r= 0.250 |
| IL-8  x  IPAQ min/week | p= 0.196  r= 0.257 | p= 0.085  r= 0.326 | p= 0.596  r= 0.171 |
| IL10/IL-6  x  IPAQ min/week | p= 0.100  r= 0.323 | p= 0.842  r= 0.039 | p= 0.898  r= -0.041 |
| IL-10/ TNF-α  x  IPAQ min/week | p= 0.234  r= 0.237 | p= 0.552  r= -0.115 | p= 0.086  r= -0.516 |
| IL-10/IL-8  x  IPAQ min/week | p= 0.593  r= -0.108 | p= 0.168  r= -0.263 | p= 0.748  r= 0.103 |

Table S2. Pearson’s coefficient correlation (r) and significance (p) analysis of cytokines or cytokine ratio.

Note: IL, interleukin; IPAQ, International Physical Activity Questionnaire; TNF-α, alpha tumor necrosis factor.
